# Understanding enhanced rotational dynamics of active probes in rod suspensions†

**DOI:** 10.1039/d2sm00583b

**Published:** 2022-08-05

**Authors:** N. Narinder, M. F. Bos, C. Abaurrea-Velasco, J. de Graaf, C. Bechinger

**Affiliations:** Fachbereich Physik, Universität Konstanz 78464 Konstanz Germany clemens.bechinger@uni-konstanz.de; Institute for Theoretical Physics, Center for Extreme Matter and Emergent Phenomena, Utrecht University, Princetonplein 5 Utrecht 3584 CC The Netherlands

## Abstract

Active Brownian particles (APs) have recently been shown to exhibit enhanced rotational diffusion (ERD) in complex fluids. Here, we experimentally observe ERD and numerically corroborate its microscopic origin for a quasi-two-dimensional suspension of colloidal rods. At high density, the rods form small rafts, wherein they perform small-amplitude, high-frequency longitudinal displacements. Activity couples AP-rod contacts to reorientation, with the variance therein leading to ERD. This is captured by a local, rather than a global relaxation time, as used in previous phenomenological modeling. Our result should prove relevant to the microrheological characterization of complex fluids and furthering our understanding of the dynamics of microorganisms in such media.

## Abstract 2

Experiment and simulation reveal how short-time dynamical modes in a dense 2D suspension of colloidal rods lead to enhanced rotational diffusion of an embedded self-propelled probe: activity couples the probe's rotation to fluctuations in rod rafts.

## Graphical abstract

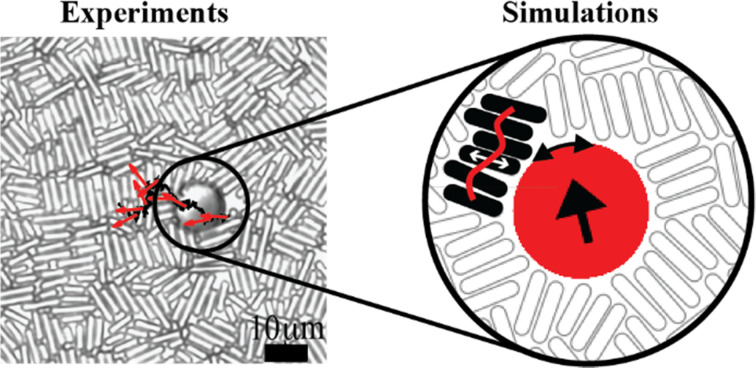

## 1 Introduction

Synthetic active particles (APs) which self-propel in liquids have received considerable interest for the following reasons. On the one hand, they serve as simple model systems to understand the motion of microorganisms and, on the other hand, they hold potential as micromachines and as drug delivery devices.^1–3^ Because their motion strongly depends on the properties of the surrounding liquid, APs can additionally serve as microrheological probes capable of characterizing the properties of the swimming medium.^4,5^ Opposed to Newtonian, *i.e.*, purely viscous fluids, where the behavior of APs has been studied in great detail,^3,6^ much less is known when these particles move in complex fluids, *e.g.*, polymer and micellar solutions or dense colloidal suspensions. The fluid's nonlinear rheological properties can lead to a strongly modified AP translational dynamics as observed in experiments^7,8^ and confirmed by theory.^9–11^ In addition, the AP angular dynamics can be significantly changed by the non-Newtonian nature of their surroundings. For example, in a viscoelastic medium, a drastically enhanced rotational diffusion (ERD) coefficient^4,5,12,13^ and even a persistent circular motion of APs has been observed.^14^ Remarkably, such intriguing behavior is present even at small self-propulsion velocities, where the rheological response is linear.^12,15^

Despite phenomenological explanations^4,14^ and numerical models,^5,13,16^ a comprehensive understanding of the above behaviors is still missing. In particular, it is not clear whether ERD can be understood (i) by treating the fluid as an effective medium with large—compared to Newtonian fluids—stress-relaxation time,^4,14^ or (ii) by explicitly considering the mesoscopic particulate structure of the swimming medium.^5,13^ To fully exploit the potential of APs as microrheological probes, however, a detailed insight into their coupling to complex fluids is mandatory.

Here, we investigate the motion of a light-driven AP in a polydisperse quasi-two-dimensional (quasi-2D) suspension of colloidal rods. Compared to previous experiments with a dense spherical colloid suspension as the swimming medium, the use of rods allowed us to unlock a new mode of fast, local structural dynamics that we were able to observe directly using optical microscopy. Combining experiments and simulations, we conclude that ERD emerges from the variance in the short-range (contact) interactions between the surrounding and the AP, mediated by it's propulsive displacement. This variance provides a natural link to the material properties of the suspension, as known from passive microrheology.^17–19^

## 2 Experimental methods

Our experiments were performed in a thin sample cell containing a polydisperse suspension of silica rods (Nippon Electric Glass Co. Ltd.) with mean length *l* = 9.8 μm (42% variance) and width *w* = 1.5 μm (2.5% variance). The rods were suspended in a critical mixture of water and propylene glycol *n*-propyl ether (PnP) which was kept 7 °C below its critical temperature, *T*_c_ = 31.9 °C.^20,21^ Owing to their small gravitational height (≈15 nm), rods sediment to the bottom of the sample cell, where they were observed to perform translational and orientational Brownian motion. Polydispersity strongly suppressed crystallization of the rods, even at the highest area fraction *φ* = 0.94 used in this work. Instead, we found a two-fold glass transition—expected for oblong particles^22,23^—by analyzing the orientational correlation and the self-intermediate scattering function for a range of *φ*, see Section S1 of ESI.† Brownian rod reorientation slowed down with increasing *φ*, eventually freezing out at *φ*^*θ*^_g_ ≈ 0.88 and resulting in an orientational glassy state. Further increasing *φ* led to the additional slow down and freezing of rod displacements at *φ*^*T*^_g_ ≈ 0.92; marking the translational glass transition in our system. The properties of the rod suspension are further detailed in Section S1 of ESI.†

A small amount of monodisperse APs were added to the suspension. They were made from silica spheres (diameter 13.7 μm) that were half-coated with a light absorbing 80 nm carbon layer. Under laser illumination (*λ* = 532 nm) and in presence of the water and propylene glycol fluid, the APs are asymmetrically heated leading to an intensity-dependent active motion with velocity *v* owing to a local demixing of the solvent.^12^ The presence of rods at intermediate to high *φ* strongly affects the AP's velocity, therefore propulsion velocities *v*_0_ given in this work are quantified according to the value at *φ* = 0. Gravity and hydrodynamic interactions with the top and bottom surface of the sample cell render the translational and rotational motion of our APs to be confined to two dimensions, as commonly observed for active system.^24,25^

## 3 Experimental results

**Fig. 1:**
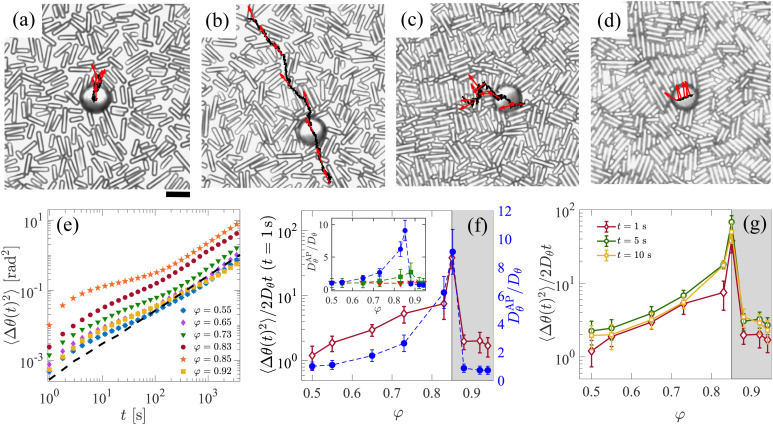
Rotational diffusion enhancement (ERD) of an active particle (AP) in a dense suspension of colloidal rods with area fraction *φ*. (a–d) Microscopy images showing: (a) an inactive probe (*v*_0_ = 0 μm s^−1^) at *φ* = 0.55; an AP (*v*_0_ = 0.7 μm s^−1^) at (b) *φ* = 0.55, (c) *φ* = 0.85, and (d) *φ* = 0.92. The black curve shows the AP's trajectory measured over 1500 s and red arrows indicate its orientation. The scale bar is 10 μm. (e) The AP's mean squared angular displacement (MSAD;〈Δ*θ*(*t*)^2^〉) as a function of time *t* for various *φ*. The dashed line represents the MSAD for an inactive probe at *φ* = 0.85. (f) Left axis and maroon diamonds: the *t* = 1 s value of the AP's MSAD as a function of *φ*; this is normalized by the equivalent (*φ* = 0) MSAD of an inactive probe using the associated long-time diffusion coefficient *D*_*θ*_. Right axis and blue circles: The AP's long-time rotational diffusion coefficient *D*^AP^_*θ*_ as a function of *φ* (normalized by *D*_*θ*_). Inset: *D*^AP^_*θ*_ as a function of *φ* for various self-propulsion speeds: *v*_0_ = 0 (orange triangles), *v*_0_ = 0.3 μm s^−1^ (green squares) and, *v*_0_ = 0.7 μm s^−1^ (blue circles). (g) The value of AP's MSAD at various times *t* (see the legend) for different *φ*. The gray-shaded region in (f) and (g) corresponds to *φ* ≥ 0.85.

Fig. 1(a and b) compares trajectories of an inactive (*v*_0_ = 0 μm s^−1^) and active (*v*_0_ = 0.7 μm s^−1^) Janus particle over a time interval of 1500 s; both were suspended in a rod background with *φ* = 0.55. Due to the AP's large size and large (*φ* = 0) orientational diffusion time *τ*_*θ*_ ≈ 7900 s, their trajectories are nearly straight. As expected, the AP's translational motion decreases upon increasing *φ* to 0.85 (Fig. 1c) and 0.92 (Fig. 1d), respectively. Opposed to this and indicated by the arrows, however, the AP's orientational dynamics reveal a pronounced non-monotonicity as a function of *φ*. The translational and rotational motion of the inactive Janus particle, and the translational motion of the AP, are characterised in detail in Section S2 of ESI.†

We quantified the AP's angular dynamics by computing the mean squared angular displacement (MSAD)〈Δ*θ*(*t*)^2^〉 = 〈|*θ*(*t* + *t*_0_) − *θ*(*t*_0_)|^2^〉, see Fig. 1(e). Above *t* ≈ 200 s and rather independent of *φ*, 〈Δ*θ*(*t*)^2^〉 is linear. This allows us to determine the AP's effective rotational diffusion coefficient *D*^AP^_*θ*_; for large *t*, 〈Δ*θ*(*t*)^2^〉 = 2*D*^AP^_*θ*_*t*. Fig. 1(f) shows *D*^AP^_*θ*_ as a function of *φ* (blue circles), which clearly exhibits a maximum around *φ* ≈ 0.85, *i.e.*, close to where the rods form an orientational glass. This behavior is similar to earlier observations with a glass background comprised of binary colloidal spheres, for which *D*^AP^_*θ*_ was also found to be largest at the corresponding glass transition. This and the increase of *D*^AP^_*θ*_ with *v*_0_ (inset Fig. 1(f)) constitute the characteristics of ERD.^4^

Remarkably, a similar *φ*-dependence as found for *D*^AP^_*θ*_ is present in the short-time properties of the MSAD. Because the MSAD is not linear for *t* ≲ 100 s, a short-time diffusion constant cannot be defined. Instead, we calculated the absolute value of the MSAD at *t* = 1 s, which we have normalized here by the corresponding value for *φ* = 0 (open diamonds in Fig. 1(f)). The trend in this quantity compares very well with that in *D*^AP^_*θ*_; a similar behavior in the MSAD is also found at other values of *t* in a range of 5 s and 10 s, see Fig. 1(g). Notably, the non-monotonic dependence on *φ* for the AP's short-time orientational behavior is not present in the short-time rotational diffusion of inactive Janus particles (Fig. S3 ESI†). This comparison suggests that the AP's propulsive motion is key to ERD.

**Fig. 2:**
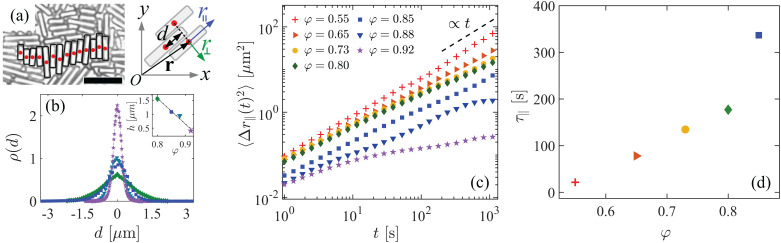
Characterizing the longitudinal fluctuation dynamics of the passive glass. (a; left) Snapshot showing a raft of rods (black highlight) at *φ* = 0.88. The red circles indicate the centers of mass. (a; right) Sketch of the rods in a raft defining the coordinates and symbols relevant to our analysis. The particle position *r⃑* = (*x*, *y*) is decomposed into components parallel (*r*_‖_) and perpendicular (*r*_⊥_) to the rod's long axis. The longitudinal displacement of the rod is characterized by *d*, which is the displacement of a rod's center from the midpoint on the line connecting the centers of the two adjacent rods. (b) The probability distribution function (PDF) *ρ*(*d*) of the longitudinal displacements *d* for different area fractions *φ* labelled with the same symbols as in (c). Inset: The half-width *h* of *ρ*(*d*) as a function of *φ*. (c) Mean squared displacement of the rod motion parallel to its long axis 〈Δ*r*_‖_(*t*)^2^〉 for various area fractions *φ*. (d) The relaxation time *τ*_‖_ associated with the longitudinal rod motion as a function of *φ*.

Our observation of a strongly enhanced orientational AP dynamics at short times and the transition toward an effective rotational diffusion around *t* ≈ 100 s, both provide important clues to the microscopic dynamics underlying ERD. We note that for *φ* ≳ 0.80, the rods form small rafts of parallel aligned particles, see Fig. 2(a), wherein they fluctuate along their long axis. We quantified this particular dynamic mode by measuring the displacement *d* of a rod's center from the midpoint on the line connecting the centers of the two adjacent rods in the raft. The averaged probability distribution functions (PDF) *ρ*(*d*) are shown in Fig. 2(b) and reveal that these fluctuations are generally small (half-width value *h* ≈ 1 μm) compared to the mean rod length *l* ≈ 9.8 μm. Nevertheless, these small longitudinal fluctuations turn out to be crucial for ERD, as shown below. As expected, *h* decreases with *φ*, but remains finite even for area fractions as large as *φ* = 0.92, *i.e.*, up to the translational glass transition.

We also computed the longitudinal MSD, that is, the mean-squared of rod displacements projected along their long axis, see Fig. 2(c). These exhibit a long-time diffusive regime with associated translational diffusion coefficients *D*_‖_ that slightly decrease with increasing *φ*. Using the mean value *h* ≈ 1 μm and *D*_‖_, we obtained a characteristic time scale for the fluctuations *τ*_‖_ = *h*^2^/*D*_‖_, which is shown in Fig. 2(d). In the presence of an AP, such longitudinal rod fluctuations should give rise to a random force on the probe. However, the time scale obtained suggests a deeper connection to the origin of ERD, as it corresponds well to the transition time from the short-time super diffusive to the long-time diffusive orientational AP dynamics, see Fig. 1(e). Note that our choice of *h* gives an approximate time scale, however, this is sufficiently accurate to make this statement. Additionally, this time scale is well separated from those associated with the glassy dynamics in our system.

Because ERD is only observed in case of active (not Brownian) probe particles, by necessity activity must be key to providing a coupling between the translational rod fluctuations and the AP's orientational dynamics. Unfortunately, the tips of the rods are partially obscured during rod-AP contact, as the spherical probe is imaged from above. This makes it difficult to resolve these interactions in experiments. We therefore performed simulations with a disc-shaped probe, which allows us to fully resolve the microscopic encounters with the rods, as discussed next.

## 4 Simulation methods

Our simulations are based on a Brownian dynamics model introduced previously.^5^ In this model, the motion of the particles and their interactions are considered in 2D, however, to better account for their shape as in experiments, 3D values of their diffusion coefficients are assumed. The rods are represented as stadiums *i.e.*, 2D-spherocylinders, which are made of a rectangle, with length *L* and width *σ*, and two disk-shaped caps of diameter *σ* (see ESI† Fig. S5(a)). Since we are mainly interested in the contact dynamics between the rods and the AP, we did not intend to incorporate all the details of the experiments.

In comparison to experiments, we used rods of shorter lengths with smaller length variation to increase the efficiency of our simulations. The stadium length *L* was drawn from a Gaussian distribution with mean 〈*L*〉 = 3*σ* and standard deviation Δ_*L*_ = 0.3*σ*. The particles interact *via* the short-ranged Weeks–Chandler–Andersen (WCA) potential with a strength of *ε* = 10*k*_B_*T* (*k*_B_ is the Boltzmann constant and *T* is the temperature). This makes the simulation system interaction-wise somewhat ‘softer’ compared to the experiment. However, this has the advantage of a considerable speed up of our simulation.^5^ The smaller Δ_*L*_ compared to experiment further improves the computational efficiency. Although these choices modify the system compared to experiments, they also allow us to highlight the robustness of the proposed physical mechanism for the observed ERD. The complete equations of motion of the rods are given in Section S3 of ESI.†

The AP is represented by a disk with diameter *σ*_AP_ = 8*σ*. The experimental AP diameter is 9*σ*, however, the effective contacts are made well below the sphere equator leading to an effective contact diameter of 
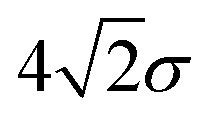
. Our disk is thus effectively larger than the one experienced by the passive rods in the experiment, but it is comparable in size. The speed of the probe is given by *v*_0_ = 100*σ*_AP_*D*_*θ*_, where *D*_*θ*_ is the free rotational diffusion coefficient of the probe. This *v*_0_ value was chosen such that the AP only weakly perturbs the structure of the surrounding rods, which is similar to the experiments. The complete equations of motion of the AP are given in ESI† Section S3. In a passive, frictionless system, there is no torque acting on the disk-like probe. We simulated the activity-induced reorientation *via* an active torque 
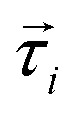
 generated by contacts between the probe and neighboring rods. Here, we used an expression that mimics rolling friction in granular systems (*e.g.*, see the work of Luding^26^) as introduced for active probes in Abaurrea-Velasco *et al.*:^5^1
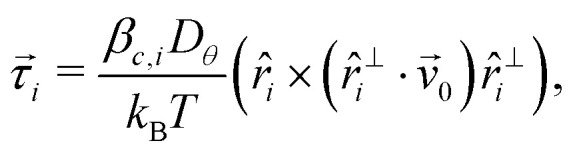
where *r̂*_*i*_ is the unit vector in the direction connecting the AP's center of mass with closest point of the *i*-th neighboring rod, *r̂*^⊥^_*i*_ is the unit vector perpendicular to this direction, and *β*_*c*,*i*_ is a coupling parameter that increases linearly from 0 to 1 with the strength of the interaction between the AP and the rod, up to a cutoff force of *F*_c_ = 20*k*_B_*T*/*σ*. We utilized this force threshold to better capture the observed rotational behavior of the AP in the experiments. That is, at lower *φ* values no significant enhancement of the rotational dynamics is observed in experiments. Enforcing this observation, we appropriately choose *F*_c_ = 20*k*_B_*T*/*σ*, which satisfies this condition. The detailed description of the choice of *F*_c_ value is further provided in Section S6 of ESI.†

In our simulations, we used a 2D square simulation box with periodic boundary conditions containing 1000 stadiums. We generated a distribution of stadium lengths for a given *φ*, and used that distribution for every initialization at that specific value of *φ*. We achieved the desired area fraction by varying the edge length *L*′ of our simulation box. For simulations with a probe particle, we placed a single disk in the box; its presence did not result in any significant change in the value of *φ*. We initialized the system by placing all stadiums (and probe) randomly in the box, after which we increased the interaction strength using power-law growth from *ε* = 0*k*_B_*T* to *ε* = 10*k*_B_*T* in approximately 10^5^ Δ*t*, where Δ*t* ≈ 2.5 × 10^−7^*D*_*θ*_^−1^ is the time step used in our simulations. The large forces and arrested dynamics present in our system near the glass transitions necessitated a relatively small time step Δ*t*. We therefore report our simulation results throughout using *D*_*θ*_^−1^ as the base physical time scale.

After initialization, we let the system equilibrate for 3.35*D*_*θ*_^−1^, after which we measured up to 50*D*_*θ*_^−1^. Although these values seem small, the ratio between the probe's translational diffusion time *τ*_T_ and rotational diffusion time *τ*_*θ*_ is 0.05, which means that we measured for 1000 translational diffusion times in total. Thus, we were able to capture the diffusive regime of an inactive probe's MSD. Lastly, it should also be noted that the self-propulsion of the AP was turned on only after the equilibration. This made it easier to achieve convergence of our initialization routine for higher *φ*.

## 5 Simulation results

First we briefly cover the passive result. Similar to the experiments, polydispersity of the simulated rods suppressed the long-range order for all considered *φ*. The passive rod system exhibits an orientational and translational ‘glass’ transition, located here at *φ*^*θ*^_g_ ≈ 0.76 and *φ*^*T*^_g_ ≈ 0.77, respectively (see ESI† Fig. S6). At sufficiently high *φ*, the rods also organized themselves in short rafts, as observed in experiments, see ESI† Fig. S6(a–c). The glass transitions occur at a lower area fractions in simulations compared to experiments. This is because we used the length scale *σ* of the WCA potential to calculate area fraction in simulations, while—due to the soft potential—the length scale on which the particles interact is larger than *σ*. This means that effectively the area fraction of the simulations is higher than the one reported here.

**Fig. 3:**
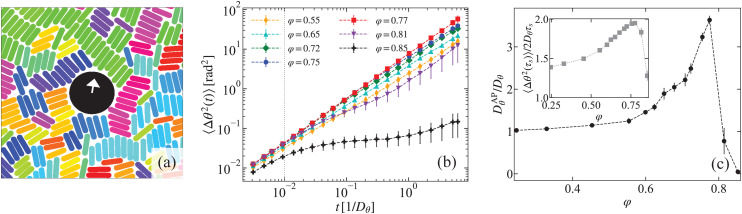
ERD of simulated AP's with area fraction *φ*. (a) Simulation snapshot of AP in rod suspension at *φ* = 0.75. The rods are colored according to their orientation. (b) The AP's MSAD (〈Δ*θ*(*t*)^2^〉) as function of time for various *φ*. (c) The AP's long-time rotational coefficient *D*^AP^_*θ*_ as function of *φ*. The inset shows the AP's short-time MSAD 〈Δ*θ*(*t*)^2^〉 evaluated at time *t* = *τ*_s_ as function of *φ*; this quantity is normalized by the associated *φ* = 0 MSAD value 2*D*_*θ*_*t*_s_ in analogy to our experimental result in Fig. 1.

Next, we studied the behavior of an AP in a rod suspension for various values of *φ*. A representative snapshot of an AP with the surrounding rods environment at *φ* = 0.75 is shown in Fig. 3(a), where the rods are colored according to their orientation. The rotational dynamics of the AP is quantified by measuring the MSAD of the probe, see Fig. 3(b). Above *t* ≈ 10^−1^*D*_*θ*_^−1^, 〈Δ*θ*(*t*)^2^〉 is linear. This allows us to determine *D*^AP^_*θ*_, as shown in Fig. 3(c). Clearly, our model captures the salient feature of the ERD. The rotational diffusion coefficient of the AP *D*^AP^_*θ*_ is (asymmetrically) peaked around *φ* ≈ 0.77 and is strongly suppressed for *φ* > 0.77. In line with the experimental findings, we also found a *φ*-dependence of the short-time angular dynamics, see the inset to Fig. 3(c), where we used the measure introduced above in Fig. 1(f). The time *τ*_s_ = 10^−3^*D*_*θ*_^−1^ is a short time scale, which is orders of magnitude smaller than the time scale over which the AP exhibits linear diffusive behavior; this can be seen in the MSAD of the AP in Fig. 3. The AP dynamics is futher detailed in Section S5 of ESI.†

**Fig. 4:**
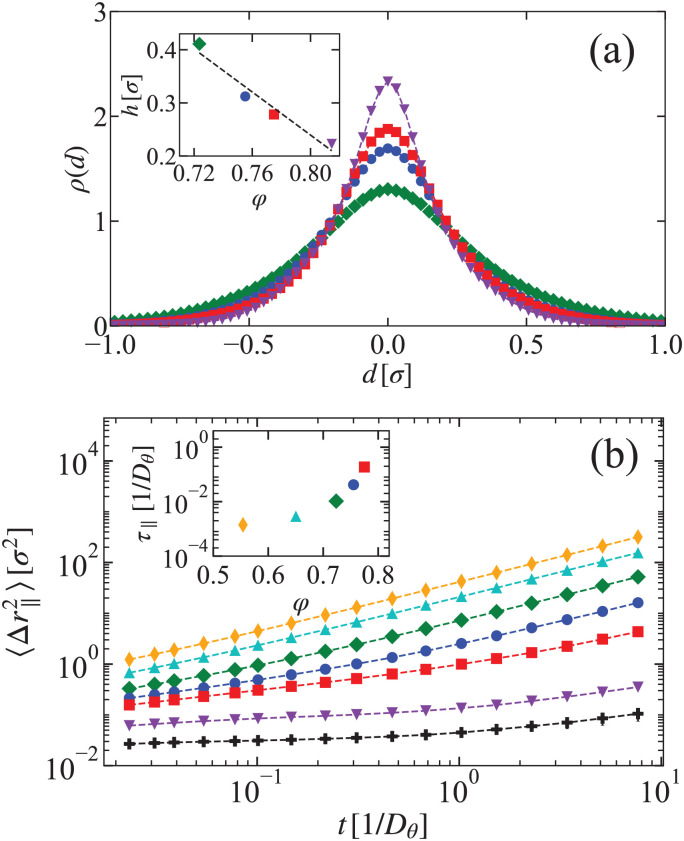
Characterizing the longitudinal fluctuating dynamics of the passive rod suspension in simulations. (a) The PDF *ρ*(*d*) of the longitudinal displacements *d* for different area fractions *φ* = 0.72 (green), 0.75 (blue), 0.77 (red), and 0.81 (purple). The inset shows the width of the distribution *h*. (b) The MSD of the rod motion parallel to its long axis 〈Δ*r*_‖_^2^〉 for various area fractions. Inset: The relaxation time *τ*_‖_ associated with the longitudinal rod motion.

Similar to experiments, we determined the time scale associated with the longitudinal rod fluctuations in simulations from the mean squared displacements of the rods along their long axis, see Fig. 4(b). From a linear fit of the form 2*D*_‖_*t*, we obtain *D*_‖_, the diffusion coefficient associated with the motion of the rods along their long axis. This provides us the time scale of the rods’ longitudinal motion: *τ*_‖_ = *h*^2^/*D*_‖_. For *h*, we used *h* ≈ 0.3*σ*, which was determined from the average width of the PDF for longitudinal rod displacements in rafts, as shown in Fig. 4(a). The obtained time scales are plotted in the inset to Fig. 4(b). As expected, *τ*_‖_ increases with increasing *φ*. At an area fraction of *φ* = 0.77 we find *τ*_‖_ ≈ 10^−1^*D*_*θ*_^−1^, which is comparable to the time scale on which the MSAD of the AP shows linear diffusion (see Fig. 3(b)).

**Fig. 5:**
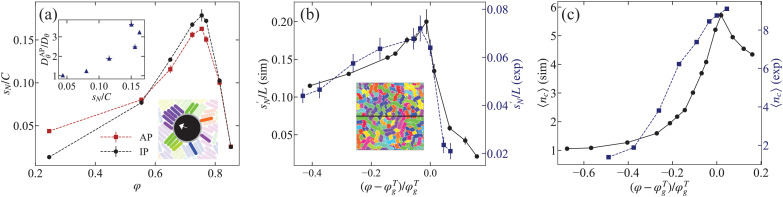
A simulated AP's ERD stems from the variance in the number of contacts it makes with the rod fluid. (a) The variance of number of rod contacts *s*_*N*_ with active probe (AP; dashed line) and inactive probe (IP; solid line) weighted by the particle's contour length *C* in simulations. The upper inset shows the correlation between the variance of the number of contacts with the AP and the ERD before the glass transition (*φ* ≤ 0.77). The lower inset shows a snapshot of the AP and its environment where all rods in contact with the probe are rendered opaque. (b) The variance over of the number of rods contacts 
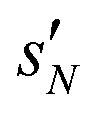
 in a passive rod suspension without probe, as measured over a line. The quantity is graphed as a function of reduced area fraction to facilitate comparison between simulations (left axis; bullets, solid black curve) and experiments (right axis; squares, dashed blue curve). The inset provides a representative example simulation snapshot at *φ* = 0.77 that shows the (black) line over which the variance was measured. (c) The average raft length 〈*n*_c_〉 as function of *φ* in simulations (left axis; bullets, solid black curve) and experiments (right axis; squares, dashed blue curve).

We subsequently investigated the rod dynamics close to the probe by measuring the number of rods *N*_c_ that make contact. Details on how we defined contact are given in Section S6 of ESI.† Irrespective of the probe's activity, we find the following: the average of *N*_c_ monotonically increases with increasing *φ*, see ESI† Fig. S8(b). However, as can be seen in Fig. 5(a), the variance of the number of contacts *s*_*N*_ normalized by the contour length of the probe *C* shows a peak that closely resembles the trend observed in *D*^AP^_*θ*_. The correlation is shown in the inset to Fig. 5(a). Note that this result has an analogy in the relation between ERD and the change in the number of neighbors as a function of *φ* for a spherical glass former.^5^

To further investigate the origin of this variance, we also measured the variance for a passive particle, see Fig. 5(a). Clearly *s*_*N*_ is not (strongly) dependent on the activity, which indicates that these fluctuations are not induced by the AP, but rather are a property of the rod suspension. Indeed, we found that such a contact variance peaks close to glass transition irrespective of the nature of the contact. For instance, we show in Fig. 5(b) the variance 
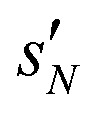
 measured along a line in a suspension of rods without a probe; the inset to Fig. 5(b) illustrates the setup. This variance 
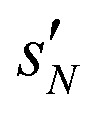
 also exhibits a maximum at the glass transition. It is likely more sharply peaked, because the average is effectively taken over a longer segment.

Intriguingly, despite the differences between experiment and simulation, a comparison of the two reveals remarkable agreement for the scaled trend in 
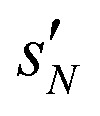
, see Fig. 5(b). We surmise from this that the peak in the variance is a generic feature of the glass transition in 2D rod suspensions. Namely, it is indicative of changes in the bulk rheological properties of these suspensions. We will return to this point in our discussion. It would be interesting to examine to what extent this feature is present in disk suspensions with glassy dynamics; this is left for future study.

The observed trends in the variance raised the question whether there is a structural change in the system at the glass transition, which is picked up by the probe through its influence on the contact variance. As discussed before, the rafts facilitate effective small fluctuations of the rods, *i.e.*, motion along their long axis. These small-scale fluctuations cause a large variance of the number of contacts, which is key for the peak of the enhanced rotational motion of the AP. Hence, we would expect the raft structures to change. We measured the average number of rods in a raft 〈*n*_c_〉, which is an easy to measure proxy for the raft structure under the assumption that the system is not too polydisperse. The results are shown in Fig. 5(c); in Section S4 of ESI,† where we explain in detail how we measured 〈*n*_c_〉.

We found that the average raft length increases when approaching the *φ*^*T*^_g_ in both experiments and simulations. Surprisingly, in simulations we found that the average raft length decreases for *φ* > *φ*^*T*^_g_, *i.e.*, 〈*n*_c_〉 peaks (sharply) at the glass transition. This strongly suggests that in the simulations there is a connection between the structure of the rod suspension, the contact variance, and AP's angular dynamics. In experiments, we found that 〈*n*_c_〉 continues to increase for *φ* > *φ*^*T*^_g_. This can be explained by the larger polydispersity present therein. A small fraction of long rods can frustrate the breaking of rafts and can even bridge rafts that would otherwise be disconnected. In addition, in experiment we observed clusters at large *φ* that are multiple rows of rods thick (see snapshot in Fig. S1(d), ESI†). These effects are also reflected in the higher value of 〈*n*_c_〉 in experiments compared to simulations for all values of *φ*.

Clearly, a decrease in experimental ERD does not coincide with a decrease 〈*n*_c_〉 in experiments. Identification of a clean correspondence between the two above the transition is hindered by the level of polydispersity. Another (yet unknown) measure of structure may reveal this connection. However, fully addressing this issue falls outside of the scope of the present work.

## 6 Discussion

The observations of the variance in the number of contacts (being valid in simulations and experiments) imply that the rod-probe contact fluctuations are always present, but that the probe's active motion is crucial to couple these to the AP's orientational dynamics. Such behavior is consistent with the coupling mechanism suggested in eqn (1), where activity comes in through the *v*_0_-dependence. In other words, only when the probe is (self-)driven relative to the (passive) rod background, fluctuating torques are generated *via* to rod-probe contacts, which eventually leads to ERD. This mechanism also explains for the first time why ERD has been also observed when *v*_0_ is not generated by activity but by particle sedimentation.^12^

To understand the relation between the observed contact variance and *D*^AP^_*θ*_, and its implications for the way in which ERD is modeled, we consider passive and active (meaning externally driven) microrheology in viscoelastic media.^27,28^ For a driven probe, collisions with colloids in the medium lead to translational fluctuations (predominantly) orthogonal to the direction of motion, which become more pronounced with increased speed and volume fraction.^29^ Transferring this concept to our active probe, strengthens the idea that ERD results from contact dynamics and strongly implies that the particulate structure of the environment should be explicitly taken into account in its modeling. Lastly, a peaked displacement response was recently found in a dense 2D suspension of colloidal spheres subjected to laser pulses.^30^ This was rationalized in terms of cooperative particle motions near the onset of glassy dynamics, which provides further support for particle-level modeling.

Turning to passive microrheology, the variance in a passive probe's rotational^31^ and translational^17–19^ displacement—induced by thermal fluctuations in the surrounding medium—was demonstrated to directly relate to the material properties of the suspension. This provides a connection between the particle and continuum framework, wherein the medium's bulk stress relaxation time is used to explain ERD.^4^ However, at the glass transition, this time is divergent and theory predicts a divergent ERD.^12^ Clearly, the time scale probed by our APs is the longitudinal (in-raft) one, which remains finite, suggesting that the probe is sensitive to more local relaxation processes. This aligns with our observation that the variance in contacts is more strongly peaked when measuring it over a long line. That is, some finite-size effects are present. It also agrees with literature findings based on microrheology of colloidal gels, wherein probes recovered the arrested dynamics but the loss and storage modulus were probe-size dependent.^32^ Mapping this dependence onto a continuum model and extracting the (local) time scale *via* that route is left for future study.

The following unifying picture now emerges. ERD always results from microscopic contact variation between the AP and its surrounding. At small AP sizes compared to the typical length scale of the surrounding, this mechanism is well captured by our discrete particle model.^5^ With increasing AP size, this contact dynamics becomes more smooth and the relaxation information contained therein approaches that of the bulk. The latter situation captures the situation of APs in molecular viscoelastic media and thus rationalizes the application of continuum formalism under such conditions.^12,14^

## 7 Summary

We have reported on experiments and simulations of active particles in a dense suspension of colloidal rods. Minute microstructural rod fluctuations together with the motion of the active probe, generate a fluctuating torque eventually leading to ERD. These fluctuations can be related to local stress relaxation, which does not diverge at the glass transition. This rationalizes why ERD remains finite even close to the glass transition for probes comparable to the particle size comprising the background medium. Beyond demonstrating the relevance of active probes for the characterization of complex materials, our findings can be relevant to uncover the specific role of complex surroundings on the swimming behavior of (model) microorganisms.

## Author contributions

Author contributions: conceptualization, C. B.; methodology, C. B. & J. d. G.; experiment, N. N.; software, C. A. V. & M. B.; investigation, N. N. & M. B. (lead), C. A. V. (supporting); writing – original draft, N. N. & M. B.; writing – review & editing, C. B. & J. d. G.; funding acquisition, C. B. & J. d. G.; resources, C. B. & J. d. G.; supervision, C. B. & J. d. G.

## Conflicts of interest

There are no conflicts to declare.

## Supplementary Material

SM-018-D2SM00583B-s001
