# Supplementary material for: Understanding enhanced rotational dynamics of active probes in rod suspensions
Source: Soft Matter. 2022 Aug 5;18(33):6246–53. doi: 10.1039/d2sm00583b (PMC9400583; doi:10.1039/d2sm00583b)
Supplement: SM-018-D2SM00583B-s001 [file SM-018-D2SM00583B-s001.pdf]

Electronic Supplementary Material (ESI) for Soft Matter.
This journal is © The Royal Society of Chemistry 2022

Electronic Supplementary Information: Understanding Enhanced Rotational Dynamics of Active Probes in Rod Suspensions

N. Narinder,[1] M.F. Bos,[2] C. Abaurrea-Velasco,[2] J. de Graaf,[2] and C. Bechinger[1]

[1] *Fachbereich Physik, Universität Konstanz, 78464 Konstanz, Germany*
[2] *Institute for Theoretical Physics, Center for Extreme Matter and Emergent Phenomena, Utrecht University, Princetonplein 5, Utrecht 3584 CC, The Netherlands*

S1. CHARACTERIZATION OF THE EXPERIMENTAL ROD SUSPENSION

This section describes the characterization of the rod suspensions studied in our experiments. We used silica rods with mean length $\langle L \rangle = 9.8\ \mu$m and width $\langle \sigma \rangle = 1.5\ \mu$m, respectively. The rods were highly polydisperse along the long axis with a coefficient of variation of 42%. The coefficient of variation along the short axis was 2.5%. A monolayer of the rods was confined to a thin sample cell, where they exhibit a quasi two-dimensional (quasi-2D) translational and orientational motion within the sample plane. We varied the density of rods in the suspension to control the area fraction $\varphi = \sum_{i=1}^{N} L_i\, \sigma_i / A$; N and A being the total number of rods in focus and the total area of the field of view, respectively. Typical snapshots of rods at different area fractions φ are shown in Fig. S1. At higher area fractions ($\varphi \gtrsim 0.80$), the rods form rafts, where several rods align parallel to each other, see Fig. S1(c) and (d).

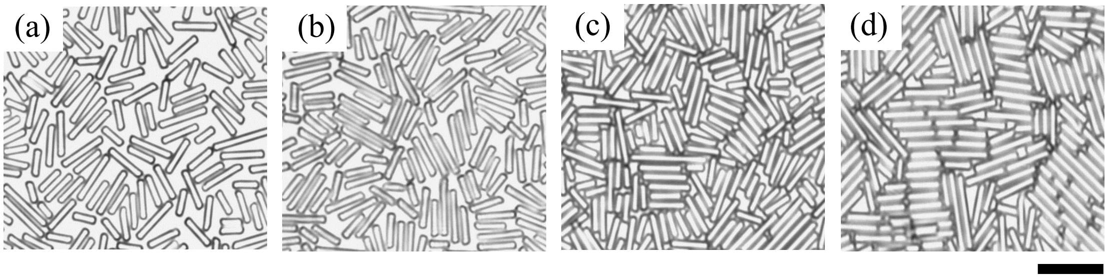

FIG. S1. Snapshots from video microscopy of the quasi-two-dimensional system of rods with mean length and width 9.8 μm and 1.5 μm, respectively, with corresponding coefficients of variation (CV%) of 40% and 2.5%. The frames show various area fractions φ: (a) $\varphi = 0.40$, (b) $\varphi = 0.55$, (c) $\varphi = 0.85$, and (d) $\varphi = 0.94$. The scale bar corresponds to 10 μm.

Figure S2(a) shows the self-intermediate scattering function (SISF) of the rods

$$F_{\mathrm{s}}(q_m, t) = \frac{1}{N} \left\langle \sum_{j=1}^{N} e^{i \boldsymbol{q}_m \cdot [\boldsymbol{r}_j(t) - \boldsymbol{r}_j(0)]} \right\rangle, \tag{S1}$$

which characterizes the translational dynamics. Here, $\boldsymbol{q}_m$ is the scattering vector and $\boldsymbol{r}_j(t)$ is the particle position at time t. The sample is isotropic and hence we can consider the SISF as a function of the vector length $q_m = |\boldsymbol{q}_m|$. We used a value of $q_m = 1.12\ \mu\mathrm{m}^{-1}$ to graph the SISFs, which is where we locate the first peak in the structure factor.

The translational relaxation time τ_{T} is defined as the time after which $F_{\mathrm{s}}(q_m, t)$ is reduced by a factor $1/e$, and this time is plotted in Fig. S2(c). The area fraction at which the glass transition takes place φ_g, is obtained by fitting the data with the following function [1]:

$$\tau_{\mathrm{T}} = (\varphi_g^{\mathrm{T}} - \varphi)^{-\gamma_{\mathrm{T}}}, \tag{S2}$$

that captures the divergence near the transition point. From the fit, we obtained $\varphi_g^{\mathrm{T}} = 0.92 \pm 0.01$ and $\gamma_{\mathrm{T}} = 2.56 \pm 0.07$. The corresponding fitted trend is given by the solid line Fig. S2 (c).

Similarly, orientational relaxation is characterized by the orientational correlation function

$$L_n(t) = \frac{1}{N} \left\langle \sum_{j=1}^{N} \cos n[\theta_j(t) - \theta_j(0)] \right\rangle \tag{S3}$$

where n is an integer and $\theta_j(t)$ is the angle of the rod with respect to the x-axis. We show the orientational correlation function $L_n(t)$ for $n = 4$ — the transition is independent of the n choice, see Ref. [2], which also provides a justification

of the use of $n = 4$ — in Fig. S2(b) and the dependence of the corresponding relaxation time (also defined in terms of the decay by $1/e$) on φ in Fig. S2(d). The orientational relaxation time τ_θ fits well to the function

$$\tau_\theta = (\varphi_g^\theta - \varphi)^{-\gamma_\theta}, \tag{S4}$$

near the orientational glass transition, see Fig. S2(d). From the divergence in the data, we locate the transition at an area fraction $\varphi_g^\theta = 0.88 \pm 0.01$ and $\gamma_\theta = 2.40 \pm 0.02$.

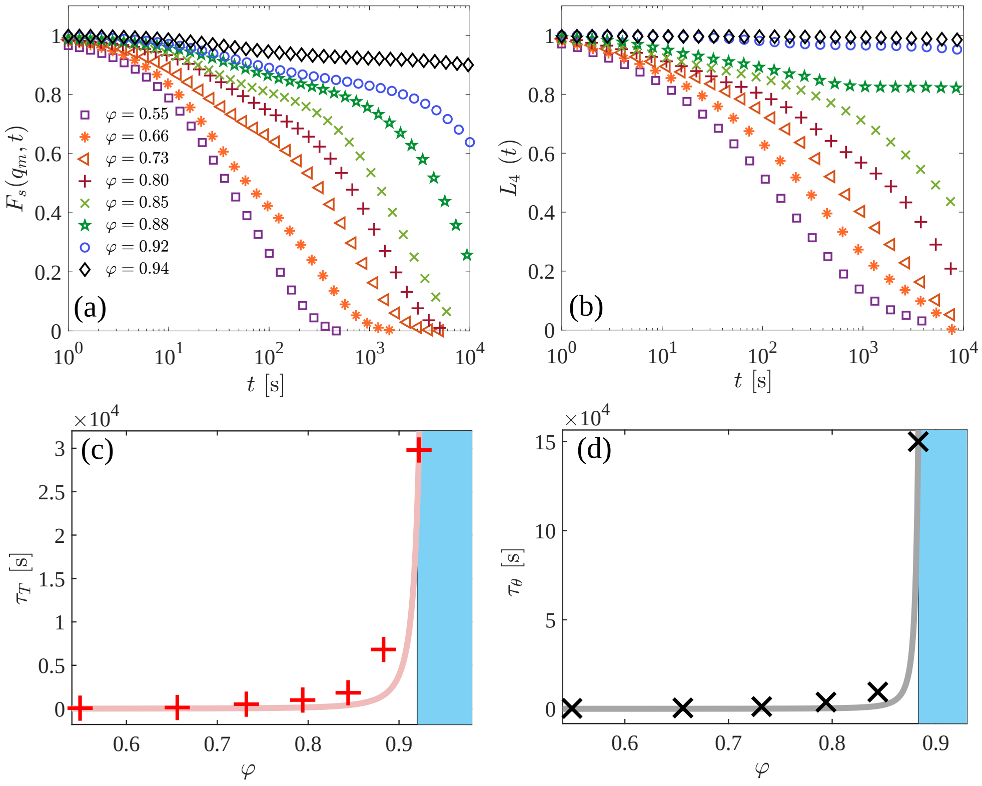

FIG. S2. (a) The self-part of intermediate scattering function $F_\mathrm{s}(q_m, t)$ as a function of time t at wave number $q_m = 1.12\ \mu\mathrm{m}^{-1}$ for different area fractions φ of the rods suspension. (b) The orientational correlation function $L_4(t)$ as a function of time for the same φ as in (a). (c) The translational relaxation time τ_T as a function of φ; τ_T is defined from $F_\mathrm{s}(q_m, t)$ as the $1/e$ decay time. The solid line corresponds to the fit $\tau_\mathrm{T} = (\varphi_g^\mathrm{T} - \varphi)^{-\gamma_\mathrm{T}}$. (d) The orientational relaxation time τ_θ as a function of φ, similarly defined from $L_4(t)$. The solid line corresponds to the fitting function $\tau_\theta = (\varphi_g^\theta - \varphi)^{-\gamma_\theta}$.

S2. THE BEHAVIOR OF THE JANUS PARTICLES IN EXPERIMENT

Figure S3 shows the mean squared displacements characterizing the behavior of an *inactive* probe in our rod suspension. That is, a carbon-coated Janus sphere with diameter 13.7 μm (in the caption, a is the radius), which is *not* illuminated. We note that the translational mean squared displacement (MSD) shows caging features, due to the glassy nature of its surroundings, as also commented on in the main text. The mean squared angular displacement (MSAD) of the probe is, however, essentially independent of the rod area fraction φ.

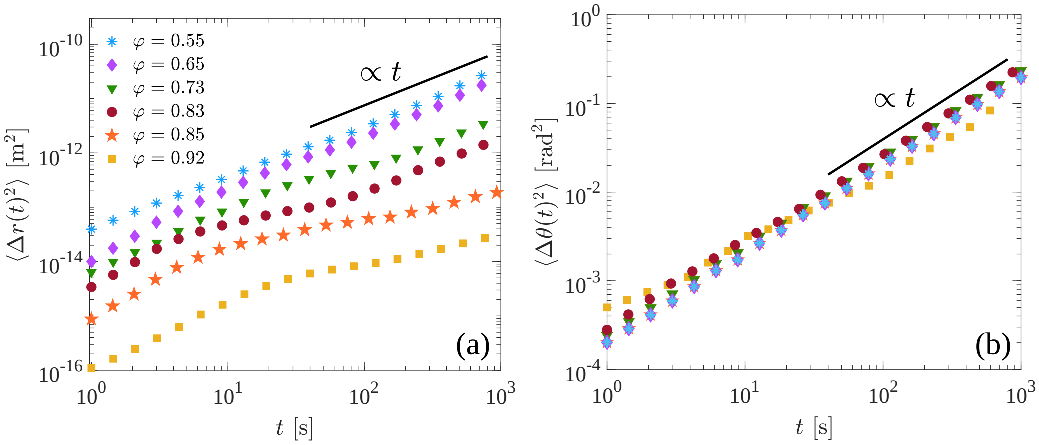

FIG. S3. Mean squared displacements of an *inactive* spherical particle (mean diameter $2a = 13.7$ μm) embedded in our rod suspension for various area fractions φ. (a) Translational mean squared displacement (MSD) $\langle \Delta r(t)^2 \rangle$ as a function of time t. (b) The corresponding mean squared angular displacement (MSAD) $\langle \Delta\theta(t)^2 \rangle$. In both panels, the solid, black lines serve as guides to the eye and show the diffusive $\propto t$ trend.

Figure S4 shows instead the behavior of the *active* particle, *i.e.*, when it is subjected to laser illumination with $\lambda = 532$ nm to make it self-propel. As in the main text, the self-propulsion speeds v_0 are those for a probe that moves in a suspension without rods ($\varphi = 0$); the effective speed at $\varphi > 0$ is lower and left unspecified.

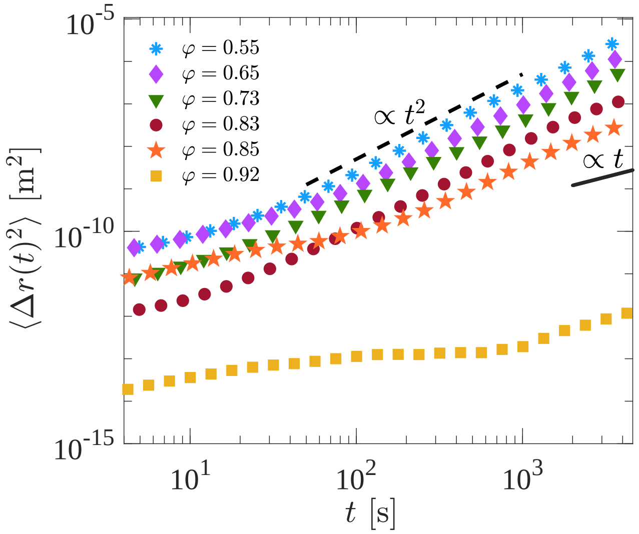

FIG. S4. (a) MSD of a spherical *active* particle (AP; identical mean diameter $2a = 13.7$ μm and moving with velocity $v_0 = 0.7$ μm s^{-1}) embedded in our rod suspension at various area fractions φ. The black (solid and dashed) lines serve as guides to the eye and show the diffusive $\propto t$ and ballistic $\propto t^2$ trends, respectively, that are typically found in active suspensions. Note that we are considering the short time trends here, there is another diffusive regime at much longer time.

S3. SIMULATION MODEL

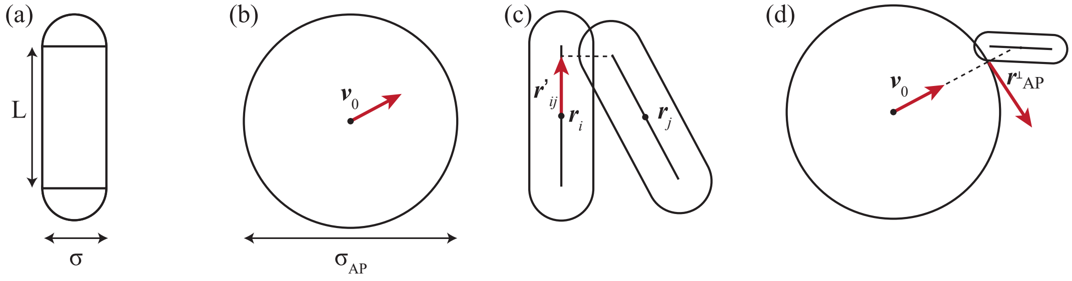

FIG. S5. Schematic of particles and their interactions in simulations. (a) Stadium (quasi-2D confined spherocylinder) with width σ and length L. (b) Spherical active probe (AP), represented here as a disk in our quasi-2D simulation with diameter σ_{AP} and self-propulsion velocity $\boldsymbol{v}_0$. (c) The arm $\boldsymbol{r}'_{ij}$, due to interactions between particle i and j, used in the torque expression. (d) The arm of the torque on the AP $\boldsymbol{r}^{\perp}_{\mathrm{AP}}$, due to the interaction with a rod.

In our simulations, we modeled the experimental systems as follows. The rods were represented as quasi-2D spherocylinders, see the schematic in Fig. S5(a); since our simulations are technically fully 2D, the correct term for our particle shape is stadiums and we will refer to them as such henceforth. This shows that a stadium consist of a rectangle and two disk-shaped caps. The width of the stadium is given by σ, which is also the diameter of the disk-shaped caps. The AP was modelled as a quasi-2D disk in simulations, as shown in Fig. S5(b).

A. Equations of Motion for the Stadiums

The motion of and the interactions between all particles are two dimensional (2D). The i-th stadium is described by the position of its center of mass $\boldsymbol{r}_i$ and orientation θ_i with respect to the x-axis. We split a stadium's velocity $\boldsymbol{v}_{i,\mathrm{st}}$ in a component parallel and perpendicular to its orientation according to

$$\boldsymbol{v}_{i,\mathrm{st}} = \boldsymbol{v}^{\perp}_{i,\mathrm{st}} + \boldsymbol{v}^{\parallel}_{i,\mathrm{st}}, \tag{S5}$$

where the superscripts $\perp$ and $\parallel$ denote the component of the perpendicular and parallel components. The associated orientational dynamics is described trough the angular velocity $\omega_{i,\mathrm{st}}$. Together, this leads to the three equations of motion that describe the entire stadium dynamics:

$$\boldsymbol{v}^{\perp}_{i,\mathrm{st}} = \frac{D^{\perp}}{k_B T}\sum_j \boldsymbol{F}^{\perp}_{ij} + \sqrt{2D^{\perp}}\boldsymbol{\xi}^{\perp}_i(t)\,; \tag{S6}$$

$$\boldsymbol{v}^{\parallel}_{i,\mathrm{st}} = \frac{D^{\parallel}}{k_B T}\sum_j \boldsymbol{F}^{\parallel}_{ij} + \sqrt{2D^{\parallel}}\boldsymbol{\xi}^{\parallel}_i(t)\,; \tag{S7}$$

$$\boldsymbol{\omega}_{i,\mathrm{st}} = \sum_j \boldsymbol{\tau}_{ij} + \sqrt{2D^{\theta}_{\mathrm{st}}\boldsymbol{\xi}^{\theta}_i} \times \hat{\boldsymbol{e}}_{\mathrm{i,st}}(t), \tag{S8}$$

where $\hat{\boldsymbol{e}}^{\perp}_{i,\mathrm{st}}$ is the unit vector perpendicular to the stadium's orientational unit vector $\hat{\boldsymbol{e}}_{i,\mathrm{st}} = (\cos\theta_i, \sin\theta_i)$. Particle i interacts with neighbouring particle j *via* the interaction force $\boldsymbol{F}_{ij}$ and torque $\boldsymbol{\tau}_{ij}$. In the above equations, T denotes the temperature of the system and k_B the Boltzmann constant. The random forces $\boldsymbol{\xi}^*_i$ account for fluid-medium-induced Brownian fluctuations and have a white-noise spectrum: $\langle \boldsymbol{\xi}^*_i \rangle = \boldsymbol{0}$, and $\langle \boldsymbol{\xi}^*_i(t) \otimes \boldsymbol{\xi}^*_j(t') \rangle = \underline{I}\delta_{ij}\delta(t-t')$, with $* = \theta$ or the translational two vector, which can be decomposed to obtain the parallel and orthogonal components; $\otimes$ denotes the dyadic product; $\underline{I}$ represents the 2D identity matrix; δ_{ij} is the Kronecker delta function; and $\delta(t-t')$ denotes the Dirac delta function. The diffusion coefficient of a stadium differs along its long and short axis and these are given by

$$D^{\parallel} = \frac{k_B T}{2\pi\eta L_i}\left(\log\frac{L_i}{\sigma} + \nu_{i,\parallel}\right) \tag{S9}$$

and

$$D^{\perp} = \frac{k_B T}{2\pi\eta L_i}\left(\log\frac{L_i}{\sigma} + \nu_{i,\perp}\right), \tag{S10}$$

respectively, where η denotes the viscosity of the fluid. For the constants $\nu_{i,\perp}$ and $\nu_{i,\parallel}$ we used $\nu_{i,\perp} = 0.839 + 0.185\sigma/L_i + 0.233(\sigma/L_i)^2$ and $\nu_{i,\parallel} = -0.207 + 0.980\sigma/L_i - 0.133(\sigma/L_i)^2$, as numerically determined by Ref. [3]. The rotational diffusion of the rod is given by

$$D^{\theta}_{\mathrm{st}} = \frac{3k_b T}{\pi\eta L_i^3}\left(\log(L_i/\sigma) + \delta_{i,\perp}\right), \tag{S11}$$

where $\delta_{i,\perp} = -0.662 + 0.917\sigma/L_i - 0.050(\sigma/L_i)^2$ is a constant determined numerically by Ref. [3].

B. Forces and Torques acting on the Particles

To obtain the forces and torques, we assume the rods and probe are nearly hard objects, which interact *via* the short-ranged, repulsive Weeks, Chandler and Anderson (WCA) potential

$$U_{\mathrm{WCA}}\left(d_{ij}\right) = \begin{cases} 4\epsilon\left[\left(\frac{\sigma_{ij}}{d_{ij}}\right)^{12} - \left(\frac{\sigma_{ij}}{d_{ij}}\right)^{6}\right] + \epsilon & \text{if } d_{ij} \leq 2^{1/6}\sigma_{ij} \\ 0 & \text{if } d_{ij} > 2^{1/6}\sigma_{ij} \end{cases}, \tag{S12}$$

where $\sigma_{ij} = (\sigma_i + \sigma_j)/2$ is the averaged particle width of the two particles (labelled i and j) involved. Note, we chose to write this down using variable widths, such that the equation may be generalized to stadium-disk interactions. The separation distance between the two particles is given by $\boldsymbol{d}_{ij}$ and has associated length d_{ij}. For stadiums d_{ij} is a function of both their centers of mass and their orientation. We used the method described in Ref. [4] to calculate this distance. We chose the interaction strength $\epsilon = 10\,k_B T$, which enabled a reasonable time step for resolving the dynamics in our simulations.

The anisotropic shape of the stadiums allows them to experience a torque when they interact with neighboring particles j. The torque generated on stadium i due to the force $\boldsymbol{F}_{ij}$ exerted by a neighboring stadium or disk-like probe is

$$\boldsymbol{\tau}_{ij} = \boldsymbol{r}'_{ij} \times \boldsymbol{F}_{ij}, \tag{S13}$$

where $\boldsymbol{r}'_{ij}$ is the lever-arm vector of rod i as shown in Fig. S5(c), which connects the center of mass of rod i to the point where $\boldsymbol{F}_{ij}$ acts.

C. Equations of Motion for the (Active) Disk

Turning to the dynamics of our (in)active probe particles — there is at most a single one in a simulation — we modeled these using the overdamped Langevin equations for the probe's translational and angular velocity:

$$\boldsymbol{v}_{\mathrm{AP}} = \frac{D_{\mathrm{T}}}{k_B T}\sum_j \boldsymbol{F}_{\mathrm{AP}j} + v_0\hat{\boldsymbol{e}}_{\mathrm{AP}}(t) + \sqrt{2D_{\mathrm{T}}}\boldsymbol{\xi}_{\mathrm{AP}}(t); \tag{S14}$$

$$\boldsymbol{\omega}_{\mathrm{AP}} = \sum_j \boldsymbol{\tau}_{\mathrm{AP}j} + \sqrt{2D_\theta}\boldsymbol{\xi}^{\theta}_{\mathrm{AP}} \times \hat{\boldsymbol{e}}_{\mathrm{AP}}(t). \tag{S15}$$

Here, $D_{\mathrm{T}} = k_B T/(3\pi\eta\sigma_{\mathrm{AP}})$ and $D_\theta = k_B T/(\pi\eta\sigma^3_{\mathrm{AP}})$ are the free translational and rotational diffusion coefficient coefficients of the probe, respectively. Again, we used 3D expressions for these quantities (those of a sphere), for the aforementioned reasons. The two random forces $\boldsymbol{\xi}_{\mathrm{AP}}$ and $\boldsymbol{\xi}^{\theta}_{\mathrm{AP}}$ have the same Gaussian property as that of the stadiums. The forces $\boldsymbol{F}_{\mathrm{AP}j}$ between an AP and surrounding rods (indexed j) are computed using Eq. (S12). Activity comes into the equations in two places. (i) Directly into the translation *via* the second term on the right-hand side of Eq. (S14). This term captures persistent motion in the direction the probe is pointing; indicated here using the unit vector $\hat{\boldsymbol{e}}_{\mathrm{AP}}$. (ii) Indirectly *via* the *active* torque $\boldsymbol{\tau}_{\mathrm{AP}j}$, which we will discuss next in section S3 D. The propulsion speed of the disk was set to $v_0 = 100\sigma_{\mathrm{AP}}D_\theta$, with D_θ as above. This proved sufficiently low a self-propulsion to prevent significant distortion of the passive stadium surrounding.

D. Generation of the Active Torque

In a passive, frictionless system, there is no torque acting on the disk-like probe. We simulated the activity-induced reorientation via an *active* torque $\boldsymbol{\tau}_{\mathrm{AP}j}$ generated by contacts between the probe and neighboring rods. Here, we used an expression that mimics rolling friction (see Ref. [5]) as introduced for active probes in Abaurrea-Velasco *et al.* [6]:

$$\boldsymbol{\tau}_{\mathrm{AP}j} = \frac{\beta_{c,j} D_\theta}{k_B T} \left(\hat{\boldsymbol{r}}_{\mathrm{AP}j} \times \left(\hat{\boldsymbol{r}}^{\perp}_{\mathrm{AP}j} \cdot \boldsymbol{v}_0 \right) \hat{\boldsymbol{r}}^{\perp}_{\mathrm{AP}j} \right), \tag{S16}$$

where $\hat{\boldsymbol{r}}_i$ is the unit vector pointing along the direction connecting the center of mass of the AP with the closest point of the rod, see the schematic in Fig. S5 (d); $\hat{\boldsymbol{r}}^{\perp}_i$ is the unit vector perpendicular to this direction. The symbol $\beta_{c,j}$ represents the coupling parameter, which we chose to depend on the magnitude of the interaction force $F_{\mathrm{AP}j}$ between the probe and rod j. When the interaction force is larger than a set cut-off force F_c, the coupling constant has a constant value of 1, below this force threshold the coupling scales linearly with the magnitude of the interaction force:

$$\beta_{c,j} = \begin{cases} \frac{F_{\mathrm{AP}j}}{F_c} & \text{if } F_{\mathrm{AP}j} < F_c \\ 1 & \text{if } F_{\mathrm{AP}j} \geq F_c \end{cases}. \tag{S17}$$

We used $F_c = 20 k_B T/\sigma$, which in practice implied that the AP only reorients slightly when it interacts with an isolated rod. Only when the system is crowded, the surrounding rods can push back on the AP sufficiently to create a significant torque and thus reorientation. We validated our choice for F_c by comparing the MSADs at an area fraction of $\varphi = 0.24$ — sufficiently low to identify single stadium-probe interactions — for three cut-off values $F_c = 0$, 10, and $20 k_B T/\sigma$. We will consider this further in section S6, once we have provided additional details on the systems's properties to ground such a discussion.

The force threshold replaced the number-of-neighbors criterion of Ref. [6], which was introduced to account for a similar effect in the bidisperse colloidal sphere background. Leading up to the completion of that study, a contact-force based criterion was also considered. However, the comparable sizes between the probe and background made it difficult to determine an appropriate cut-off value that best fit the trends observed there. In this respect, we should note that our reorientational torque model is an effective description only. We cannot make claims about the exact nature of the interaction that leads to reorientation, other than that it is sufficient for it to be short ranged. Clearly, activity is also a requisite, as evidenced by the experiments, which is captured *via* the $\boldsymbol{v}_0$ dependence in Eq. (S16).

S4. CHARACTERISTICS OF PASSIVE ROD SUSPENSION

In Fig. S6(a-c) we show snapshots of the system at an area fraction of $\varphi = 0.55$, 0.75, and 0.85. We find — similar to the experimental system — no long-range order at any area fraction. We characterized the passive stadium suspension (no added probe) using the self-intermediate scattering function $F_s(q_m, t)$ and the orientational scattering function $L_4(t)$, as given in Eq. (S1) and Eq. (S3). We used $q_m = 0.5\sigma^{-1}$, which is the location of the first peak in the structure factor. The self-intermediate scattering function is plotted in Fig. S6(d) and the orientational scattering function is plotted in Fig. S6(f). We fitted the tail of the scattering functions with a stretched exponential

$$f(t) = \exp\left[-\left(t/\tau\right)^{\beta} \right]. \tag{S18}$$

Where the fits through $F_s(q_m, t)$ give the translational relaxation times τ_{T}, as plotted in Fig. S6(e). The fits through $L_4(t)$ give the orientational relaxation times τ_θ of the passive rod system, as shown in Fig. S6(g). We located the φ for the orientational and translational glass transitions by fitting the translational relaxation times with Eq. (S2) for $\varphi > 0.6$ and the orientational relaxation time with Eq. (S4) for $\varphi > 0.6$. From these fits we obtained values of $\varphi_g^{\mathrm{T}} = 0.775$, $\gamma_T = 1.51 \pm 0.10$ and $\varphi_g^{\theta} = 0.767$, $\gamma_\theta = 1.66 \pm 0.02$, respectively.

We characterised the structure of the passive rod suspension by measuring the average number of rods in a raft $\langle n_c \rangle$. We used a cluster algorithm that identifies clusters in the system. In this algorithm, a rod is added to the cluster if the angle between the rod and a rod in the cluster is $< 10°$ and the rods are in contact in experiments or the distance between the center of mass of the stadiums is $< (\langle L \rangle + \sigma)/2$. From our clusters, we calculate the probability distribution function $P_n(n, \varphi)$ to find a raft of size n in the system at an area fraction of φ. The average n is then calculated according to

$$\langle n_c(\varphi) \rangle = \sum_{n=1}^{n=N_c} n P_n(n, \varphi), \tag{S19}$$

where N_c is the maximum cluster size found in the system.

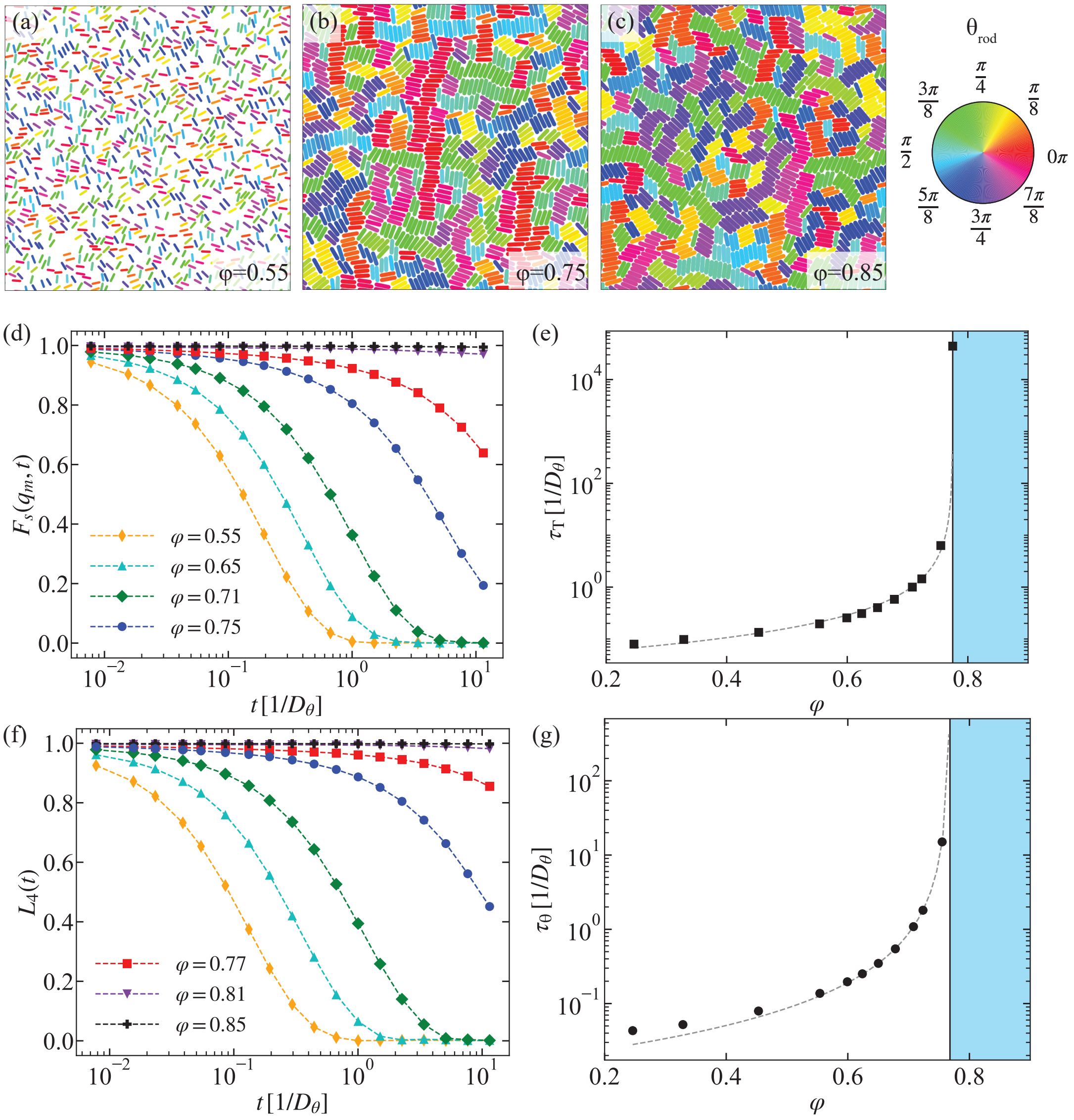

FIG. S6. Overview of the properties of a passive stadium suspension in simulations. (a)-(c) Snapshots of the system taken at area fractions of $\varphi = 0.55$, 0.75, and 0.85. The stadiums are colored according to their orientation, which is projected on the interval of $[0, \pi]$, see the color bar on the right. (d) Self-intermediate scattering function $F_s(q_m, t)$ for range of φ — see legend shared between (d,f) — as a function of time t at fixed $q_m = 0.5\sigma^{-1}$, as justified in the text. (e) Relaxation time τ_T found from fitting $F_s(q_m, t)$ with a stretched exponential, see text. The blue shaded area indicates area fractions beyond the the translational glass transition from the fit (dashed line) through the simulated τ_T. (f) Orientational correlation function $L_4(t)$ as a function of t for range of φ, see legend in (d,f). (g) The associated relaxation times τ_θ were obtained by fitting a stretched exponential to the curves in (f), see text. The blue region indicates φ values beyond the orientational glass transition, as found from the fit (dashed line) through the simulated τ_θ.

S5. CHARACTERISTICS OF THE PROBE DYNAMICS

In Fig. S7(a), we show snapshots of the AP in a system with area fraction of $\varphi = 0.75$ with time interval $0.6 \cdot 10^{-2}\, D_\theta^{-1}$. From these snapshots, it becomes clear that the AP does not move around on this time scale, but it does change its orientation. In Fig. S7(b), we show snapshots of the AP in the same system but for a larger time interval of $0.6\, D_\theta^{-1}$, where one can see that the neighborhood of the AP changes completely between snapshots. The orientation also changes considerably, as the regime of (enhanced) rotational diffusion has set in.

Figure S7(c) shows the MSD $\langle \Delta r^2(t) \rangle$ of an AP for a selection of φ. We fitted $\langle \Delta r^2(t) \rangle$ with $2D_T^{\mathrm{AP}} t$ at long times, from which we obtained D_T^{AP}, the translational diffusion coefficient of the AP. This value is shown as function of

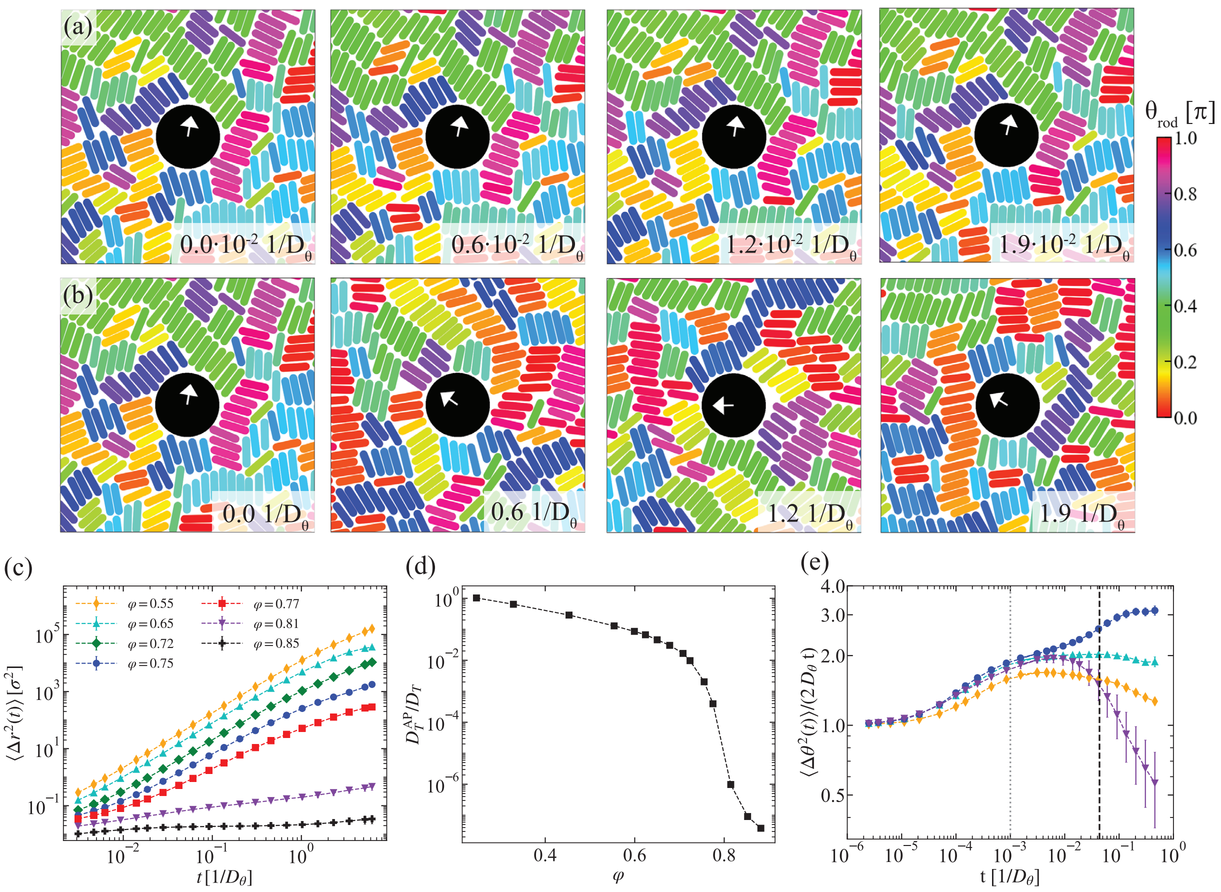

FIG. S7. Overview of dynamics of the disk-like probes used in simulations. (a,b) Snapshots in center of mass frame of the AP, where the orientation of the AP is indicated by the white arrow. The time interval is (a) $\Delta t = 0.6 \cdot 10^{-2}\, D_\theta^{-2}$ and (b) $\Delta t = 0.6\, D_\theta^{-1}$. The stadiums are colored according to their orientation, which is projected on the interval of $[0, \pi]$, see the color bar on the right. (c) The MSD $\langle \Delta r^2_{\mathrm{AP}}(t) \rangle$ for an AP as a function of time t for a selection of area fractions φ, as indicated by the legend. (d) The associated long-time translational diffusion coefficients D_T^{AP}, obtained from a linear fit to the graphs in (c) as described in the text, normalised by D_T (the free translational diffusion coefficient of an inactive disk). (e) The MSAD normalised by $2D_\theta t$, presented as a function of t, for 4 different area fractions, see the legend in (c). The grey dotted line corresponds to t_s, the contact-dynamics time as defined in the main text, and the black dashed line corresponds to $\tau_\parallel$ (obtained for $\varphi = 0.75$).

area fraction in Fig. S7(d). D_T^{AP} decreases with increasing φ, as expected for a background that exhibits arrested dynamics. Around $\varphi = 0.75$, where the AP shows a peak in the rotational diffusion, see Fig. 3(a) of the main text, the translational diffusion coefficient decreases over orders of magnitude. This makes sense, as increased rotation reduces the probe's ability to move persistently. It should be noted that the nearly concurrent translational glass transition of the stadium background will also strongly reduce the probe's ability to translate. Because the two transitions lie close together in our simulation, we cannot distinguish which of these effects dominates.

To better highlight the features of the MSADs, we present in Fig. S7(e), the MSAD divided by $2D_\theta t$. In this representation, linear diffusion is a horizontal line, where a value of 1 represents free rotational diffusion. It can be seen that for all area fractions the probe starts from free linear diffusion at very short times. This is expected to hold in general for APs at sufficiently small times [7].

At intermediate times, there is an increase in the value, which depends on the area fraction. Examining $\varphi = 0.75$ (dark blue), which lies just before the orientational glass transition, we observe two plateau-like features. We associate the first plateau with the averaging out of probe reorientations generated by individual stadium-probe contacts. We therefore expect the time scale associated with the transition to this plateau to be that of the average contact dynamics. We locate the transition at $t_s \approx 10^{-3}\, D_\theta^{-1}$ (grey dotted line) and verified that this indeed roughly corresponds to the time over which stadiums come into contact with the probe; not shown here. For φ away from the glass transition, we find that on even longer time scales, the value $\langle \Delta\theta^2(t) \rangle / (2D_\theta t)$ typically decreases (mildly below and strongly above the transition, respectively). In both cases, this can be attributed to the setting in of (sub)diffusive dynamics of the

AP's orientation. For relatively low area fractions (yellow curve), the effect of collision-based enhancement dies out, since there is a long time between reorienting collisions. This implies that the effective enhanced rotational diffusion (ERD) is relatively limited. For intermediate values of φ (cyan curve), there is little decay and collisions directly drive ERD. Turning to φ above the glass transitions (purple curve), the probe is strongly caged translationally. This implies that the AP orientational dynamics becomes equivalently caged in terms of the reorienting landscape to which the AP is subjected by contact interactions with the stadiums. This mechanism of strong ERD reduction is discussed further in Ref. [6], see also Fig. 4 of that reference.

Intriguingly, for $\varphi = 0.75$, there is a secondary increase in $\langle \Delta\theta^2(t) \rangle/(2D_\theta t)$ that plateaus at long times, where we find true ERD. We indicate the ($\varphi = 0.75$) time scale for the long-axis stadium displacement $\tau_\parallel$ — see the inset to Fig. 4(b) in the main text — as a black dashed line in Fig. S7(f). This measure corresponds well in a qualitative sense to the time where the transition to the secondary plateau is made. That is, where the orientational dynamics of the probe changes from short-time super diffusive to long-time diffusive. This is a piece of evidence in support of our conclusion that the longitudinal stadium fluctuations in their raft-like structures are key to the emergence of significant ERD.

S6. JUSTIFICATION OF THE CUT-OFF CRITERIA

In our simulations and analysis of the results, we have used two cut-off values for the force between a stadium and a disk-like probe: (i) F_c for the per-contact torque generated on the AP. This quantity was used for all simulations of the AP. (ii) The normal force between a stadium and the probe F_N, for which we consider these to be in contact. This quantity was used to generate the data in Fig. 5(a),(b) of the main text. In this section, we will discuss the effects of these cut-offs and justify the values of F_c and F_N used in our simulation.

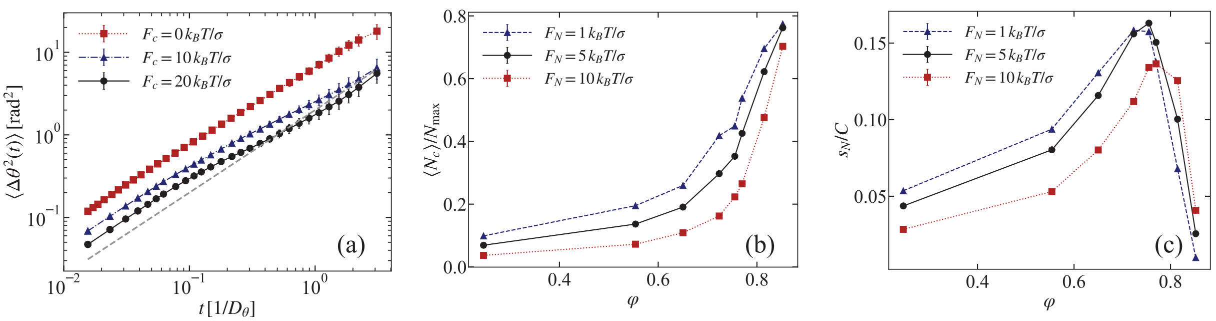

FIG. S8. Effect of the cut-offs used in our calculations. (a) The AP's MSAD $\langle \Delta\theta^2(t) \rangle$ for three different values of the cut-off F_c at an area fraction of $\varphi = 0.25$. The cut-off values are indicated in the legend and the gray dotted line represents the MSAD of freely diffusing probe. (b) Average number of contacts $\langle N_c \rangle$ between the AP and surrounding stadiums as function of φ. The value is normalized by maximum number of contacts $N_{\max}$ for 3 different values of F_N. (c) Variance of the number of contacts s_N as a function of φ for 3 different values of F_N.

As explained in section S3 D, the F_c cut-off is used to suppress torque generation on the AP that results from interaction with an isolated stadium. To determine an appropriate value for F_c, we performed simulations at a low area fraction of $\varphi = 0.25$, for which we measured the MSAD of the probe. We used a values of $F_c = 0$, 10, and $20\, k_BT/\sigma$ and compared the result to the MSAD of a freely diffusing probe, see Fig. S8(a). We found that for $F_c = 20\, k_bT/\sigma$ the passive free rotational diffusion is obtained at large times, which mimics the behavior of the AP in experiments. For lower values of F_c we find enhanced rotational diffusion, even at the low value of $\varphi = 0.25$. This justifies our choice of $F_c = 20\, k_BT/\sigma$.

Next, we consider F_N, where it is understood that a contact is present whenever the interaction force $\geq F_N$. A stadium and the probe start to interact when their separation $r < r_{\text{WCA}} = 2^{1/6}\sigma$. However, what constitutes a contact is open to consideration, as the interaction is almost hard-disk, but not quite. In addition, the generation of torque on the probe is gradually increased as r becomes smaller, up to the limiting value imposed by F_c. In Fig. 3(b) in the main text, we computed the variance of the number of contacts s_N using a contact criterion of $F_N = 5\, k_BT/\sigma$.

Figure S8(b) show what the effect of F_N is on the average number of contacts $\langle N_c \rangle / N_{\max}$ as function φ. Here, $N_{\max} = C/\sigma$ is the maximum number of contacts, with C the contour of the probe. As expected, a greater value of F_N leads to a decrease in the number of contact at all densities. However, the effect on the shape of the curve is very minor; with increasing F_N the curve becomes slightly steeper. Figure S8(c) shows the effect of F_N on the variance of the number of contacts s_N as function of φ. Similar to our result for $\langle N_c \rangle$, the overall shape is the same, however,

the exact location of the peak shifts to higher area fractions with increasing F_N. This effect suggest that the exact location of the peak of D_θ^{AP} depends the strength of the contact and how often the contact occurs.

Combining both results, we chose the value $F_N = 5\,k_B T/\sigma$ to be most representative of the trend observed in experiment. This comes with the caveat that there are significant differences in the properties of the underlying surrounding suspension between the simulations and experiment. Thus, our matching is only qualitative.

[1] W. Gotze and L. Sjogren, *Rep. Prog. Phys.*, 1992, **55**, 241.
[2] Z. Zheng, F. Wang, Y. Han *et al.*, *Phys. Rev. Lett.*, 2011, **107**, 065702.
[3] M. M. Tirado, C. L. Martínez and J. G. de la Torre, *J. Chem. Phys.*, 1984, **81**, 2047–2052.
[4] M. P. Allen, G. T. Evans, D. Frenkel, F. Greco, J. Krzystek, G. Marrucci, B. Mulder and J. von Schütz, *Adv. Chem. Phys.*, 1993, **86**, 1–166.
[5] S. Luding, *Journal of Physics: Condensed Matter*, 2005, **17**, S2623.
[6] C. Abaurrea-Velasco, C. Lozano, C. Bechinger and J. de Graaf, *Phys. Rev. Lett.*, 2020, **125**, 258002.
[7] J. R. Howse, R. A. Jones, A. J. Ryan, T. Gough, R. Vafabakhsh and R. Golestanian, *Phys. Rev. Lett.*, 2007, **99**, 048102.
